# Notes and Notices

**Published:** 1888-04

**Authors:** 


### NOTES AND NOTICES.

Nebraska State Homœopathic Society meets at Lincoln May 8th–10th.

The Hahnemannian Monthly.—Dr. Pemberton Dudley will hereafter have the entire management of this journal. *All* communications should be sent to him at southwest corner of Fifteenth and Master Streets, Philadelphia.

Lectures on the Organon.—We are glad to learn that Professor Gee will lecture weekly on the *Organon* in the post-graduate course of the Hahnemann College, of Chicago. Professor Gee is well qualified for this work, and will render good service to the students. We trust the lectures will be continued during the winter course also.

Location Desired.—By a physician, a graduate of the Homœopathic Medical College of Missouri. Is thirty-four years old, has a family, has been in practice about four years, and is a strict Hahnemannian. Would like to locate in a large town and assist an older practitioner. Address Location at this office.

What! ! * * * "The Institute is year by year becoming more and more scientific and less and less homœopathic." *Medical Advance*, January, 1888, p. 73, lines 17 and 18 from top.

We had supposed Homœopathy to be a "science," but from the above we find we are mistaken. Now let the I. H. A. come down from its perch and make more claims to be a *scientific* society. Just let the world know it is Homœopathic (with a big "H") but without a spark of science.

H. Hitchcock, M. D.

A Surgical Journal.—The *Annals of Surgery*, the only English journal published devoted exclusively to surgery, enters now upon its fourth year.

Drs. L. S. Pilcher, of Brooklyn, N. Y., and C. B. Keetley, of London, are the chief editors, assisted by most of the able surgeons of this country, as well as Europe, which is sufficient guarantee of the literary merits. We bespeak for it the co-operation of the members of the profession who are interested in progressive surgery.

J. H. Chambers & Co., St. Louis, Mo., are the publishers, and deserve great credit for undertaking to produce such an important journal as *Annals*.

For Sale.—A growing practice of three thousand dollars a year in Marysville, Yuba County, Cal., which is the county-seat, six thousand inhabitants; ten thousand inhabitants in the radius of twelve miles. Marysville is a business centre for four counties, situated in Sacramento Valley, a great fruit and grain-growing country; all kinds of fruit and berries, oranges and lemons; a mild climate, flowers in blossom every day in the year in open air. Address H. C. F., care this office.

